# Medical imaging consultation practices and challenges at public hospitals in the Amhara regional state, Northwest Ethiopia: a descriptive phenomenological study

**DOI:** 10.1186/s12913-023-09652-9

**Published:** 2023-07-24

**Authors:** Araya Mesfin Nigatu, Tesfahun Melese Yilma, Lemma Derseh Gezie, Yonathan Gebrewold, Monika Knudsen Gullslett, Shegaw Anagaw Mengiste, Binyam Tilahun

**Affiliations:** 1 Department of Health Informatics, Institute of Public Health, University of Gondar, Gondar, Ethiopia; 2 Department of Epidemiology and Biostatistics, Institute of Public Health, University of Gondar, Gondar, Ethiopia; 3 Department of Radiology, College of Medicine, Author’s Information, University of Gondar, Gondar, Ethiopia; 4 Faculty of Health and Social Sciences, University of South-Eastern Norway, Drammen, Norway; 5 Management Information Systems, University of South-Eastern Norway, Drammen, Norway

**Keywords:** Medical imaging, Radiology consultation services, Challenges, Ethiopia

## Abstract

**Background:**

Medical imaging plays a vital role in the accurate diagnosis, treatment and outcome prediction of many diseases and injuries. However, in many African countries, deserving populations do not have access to the proper medical imaging specialists’ services. As a result, clinicians continue to struggle to provide medical imaging via consultation. However, little is known about conventional referral consultation practices and their challenges. This study, therefore, aimed to explore the practice and challenges of medical imaging service consultation among health professionals and patients in the context of the Ethiopian public healthcare delivery system.

**Methods:**

Descriptive phenomenological study was employed to explore the practice of medical imaging service consultation among health professionals and patients in public hospitals of Amhara region from October 12, 2021 to December 29, 2021. Semi-structured interview guides were prepared separately for key-informant and in-depth interviews. A total of 21 participants (6 hospital managers, 4 medical directors, 4 department heads, 3 medical imaging coordinators and 4 patients) were selected using the maximum variation sampling technique. All interviews were audio-recorded, transcribed verbatim and subjected to inductive thematic analysis using Open Code 4.02 software.

**Results:**

Six major themes emerged following the thematic analysis: (1) medical image service delivery practices; (2) medical imaging consultation modalities; (3) benefits and drawbacks of the consultation modalities; (4) challenges; (5) challenge mitigation strategies; and (6) future recommendations. Image films, compact disks, and telegram apps were the consultation modalities used by the referring clinicians to send the medical images to radiologists. Frequent failure of imaging machines, delayed equipment maintenance, inadequate infrastructure, shortage of budget, lack of radiologists, and low-quality of printed image films were among the challenges influencing the medical imaging consultation service.

**Conclusions:**

This research explored onsite and referral imaging consultation practices. However, there are many challenges encountered by the referring clinicians and the radiologists during the consultation process. These challenges could potentially affect clinicians’ ability to provide timely diagnosis and treatment services which would ultimately affects patient health status and service delivery. Virtual consultation via teleradiology and enhancing clinicians’ competence through long-term and short-term trainings are recommended to improve the referral consultation practice.

**Supplementary Information:**

The online version contains supplementary material available at 10.1186/s12913-023-09652-9.

## Background

To achieve universal health coverage, medical technologies could be employed in the prevention, diagnosis, treatment, and management of medical conditions [1–3]. According to De Miranda et al. [4], medical technology is “the application of devices, procedures, and knowledge for diagnosing and treating disease for the purpose of maintaining, promoting, and restoring wellness while improving the quality of life”. Medical technology has transformed medicine by enabling doctors to study more about the human body than ever before and supporting them in clinical decision-making [4–6]. Medical imaging offers a viable solution to some of the country’s ongoing healthcare challenges, such as new emerging infectious diseases and the increasing complexity and volume of work [7–9].

Ideally, every citizen has the right to get timely access to high-quality medical treatment by trained specialists [10]. However, according to World Health Organization (WHO) estimates, half to two-thirds of the world’s population does not have adequate access to basic imaging technologies such as X-rays and ultrasound [11]. The challenge is very critical especially for low and middle income countries (LMICs), due to inadequate infrastructure, lack of imaging technology, inaccessibility, affordability, acceptability, a shortage of radiologists, migration from low-income to high-income countries, and a lack of technical experts [12, 13]. According to Kawooya et al. [14], the proportion of radiologists who migrate to other nations is significantly higher in low-income countries than in high-income countries, ranging from 2 to 35%.

Furthermore, there is a considerable discrepancy in radiology equipment distribution between low incomes to high-income countries [15]. Only 14% of low-income countries, for example, have at least one computed tomography (CT) scan per one million population, compared to 100% of high-income countries [16]. Most of the LMICs avail medical imaging machines for their health facilities through donations due to the lack of adequate budget [17]. However, around 38.3% of the donated health technology machines in developing countries are out of service because of technical and non-technical problems [18, 19]. The technical challenges have been faced from the very beginning, when requests for imaging are made [20, 21].

According to a study conducted at public hospitals in Addis Ababa (Ethiopia), 25% of imaging devices are nonfunctional due to a paucity of technicians with the necessary skills [22]. On the other hand, accessing technical experts to maintain high-quality medical imaging equipment at the health facility level remains an ongoing challenge [23]. Moreover, the number of radiologists nationally is very limited (300 radiologists to serve 118 million population) [14]. As a result of this, offering quality radiological services to patients is a challenging task. To overcome the challenges associated with patient anxiety and prolonged total examination times, medical imaging consultation is used as an alternative to properly utilize the time and skills of those limited radiologists [24]. This technique enables consulting clinicians to build confidence in the radiological report, minimize repeat imaging, and revise the diagnostic or management plan [21]. However, offering the expected services to all the deserving populations remains difficult, particularly for low- and middle-income countries [25].

To close the gaps in equity and high-quality healthcare delivery (including medical imaging service), the federal ministry of health of Ethiopia designed time-bounded guiding documents like the health information strategy (HIS) plan [26], the health sector transformation plan II (HSTP II) [27] and digital health blueprints [28]. Though medical imaging is one of the service areas demanding attention as it affects considerable portions of the primary and general hospitals clinical services, strategy and policy documents were not prepared considering evidences on radiology service consultation practices and their challenges.

Furthermore, prior studies conducted in the field of radiology [9, 29–32] paid little attention to answering questions such as: how clinicians consulted the radiological image with radiologists?, what were the available radiology service consultation practices?, what were the challenges that influenced the process of medical imaging consultation?, and what strategies could be developed to address the challenges?). So, gathering data with the help of the aforementioned inquiries could be useful to improve the provision of medical imaging services. As a result, the improved medical imaging service would benefit the referring clinicians in managing both communicable and non-communicable diseases by providing better treatment, monitoring, and predicting the outcome timely [5, 9]. Therefore, this study aimed to explore the medical imaging service consultation practice and its challenges among health professionals and patients in the context of Amhara region public hospitals, Northwest Ethiopia.

## Methods

### Study design and period

The study used descriptive phenomenological approach. This approach is helpful to provide and in-depth understanding of the lived experience of the study under investigation [33, 34]. Due to this we employed a descriptive phenomenological approach to explore the lived experiences of medical imaging consultation practices and their challenges among health professionals and patients. The approach relies on phenomenological philosophy, which entails conducting research from the standpoint of phenomenological reduction, with the goal of discovering the essential structure and meanings of subjectively lived experience [34]. The study was conducted from October 12, 2021 to December 29, 2021.

### Study setting

This study was conducted in the public hospitals found in Gondar city administration, Central Gondar and South Gondar zones of the Amhara Regional State, Northwest Ethiopia. According to the Amhara Regional State Health Bureau’s 2021/2022 annual plan performance report, the region has a total of 19 Zones including the city administrations and 238 kebeles (the lowest administrative unit) [35]. In the region, there are 98 hospitals, 917 health centers, and 3,725 public health post health facilities providing essential health services for 22,876,999 populations. Even through the population of the region was high, clinical services were provided by 241 specialists, 1307 general practitioners, and 260 radiology professionals. On the total 260 radiology professionals, 83 were radiographers (imaging technicians-diploma in radiology), 139 were radio-technologists (first degree in radiology) and 38 were radiologists.

### Sample size and sampling techniques

According to Guest et al., a minimum of 12 interviews are usually required [36]. Though we computed the initial sample size, our final sample size was determined based on the level of information saturation criterion which means when new interviews produce little or no new information to address the research question [36, 37]. As a result, since our analysis was an inductive thematic analysis, the authors of this study determined the saturation level by considering when there were no new emerging codes or themes. Thus, the study included 21 participants [17 for the key-informant interview (KII) and the rest 4 were for the in-depth interview (IDI)]. Of the total 17 KII participants, 6 were medical directors, 4 were hospital managers, 4 department heads (2 radiologists, 1 general surgeon and 1 internist) and 3 were imaging unit coordinators.

The maximum variation (heterogeneous sampling) technique was used to select the number of participants from each group and collect data on medical imaging consultation practices and their clinical and administrative challenges. According to previous research, the maximum variation sampling technique is an appropriate participant selection technique for studies involving heterogeneous groups [38].

### Participant selection

Key-informants were chosen based on their administrative responsibilities and length of hospital stay. To select participants for the KIIs and IDIs, the research investigators established an inclusion criteria before conducting the data collection. A minimum of two years of work experience was set as a criterion for KIIs because investigators believed that this was sufficient for key informants to acknowledge the hospital radiology consultation practice and associated challenges. Similarly, prior exposure to medical imaging unit visits was considered as an inclusion criteria to recruit patient participants for the in-depth interview. However, individuals who didn’t meet the illegibility criteria were excluded from the study by the principal investigator at the time of the data collection. All participants were informed about the purpose of the study and the criteria for participant selection. Each participant provided informed consent prior to the data collection.

### Participant characteristics

In our context, along with their administrative duties, medical directors (they are general practitioners by profession) and department heads of the radiology, medical surgical and internal medicine professionals spent the majority of their time providing clinical services. Furthermore, the three medical imaging unit coordinators were responsible for carrying out the requested radiographic study and transferring the images back to the requesting clinician. The consulting clinicians then perform the referral consultation to the radiologists. Despite being medical professionals, hospital managers were in charge of the hospital’s administrative aspects. Patients, on the other hand, were included based on their prior exposure to medical imaging units.

### Data collection techniques

Because the nature of our research question demanded the participation of service providers (clinicians and radiologists), administrators and patients we used KII and IDI data collection techniques. We chose KII and IDI techniques in the hopes that the diverse participants would be able to provide relevant information about traditional medical imaging consultation practices and associated challenges. There were no repeated interviews.

### Data collection tools and procedures

Separate semi-structured interview guides were developed for the KIIs and IDI participants separately (Additional file 1, Additional file 2). The interview guides were reviewed and edited by domain experts invited from health promotion and behavioral science, public health and radiology departments. The original interview guides were significantly improved by incorporating comments and suggestions from experts. A pretest was conducted on three individuals who were not involved in the study and minor modifications were made accordingly. The interview guides were prepared in English and translated into Amharic (the local language of the study area) to collect details of the data through probes.

All key-informant and in-depth interviews were conducted face-to-face by the principal investigator (AM). Key-informant interviews were conducted privately at their office at their convenience. Similarly, patients were interviewed at the hospital where they received radiology services by requesting free and convenient class. All the interviews were audio-recorded, and the interviews lasted 20 to 32 min (KII: 20 to 32 min and IDI: 23–31 min). A KII guideline document suggested that the average interview duration should be no more than 30 min [39]. Similarly, a range of 30 min to several hours is recommended for the in-depth interview [40]. Any identifying information provided by the participants was not tagged to de-identify the data to ensure confidentiality.

The questions were asked in response to participant cues to reduce respondent bias and the danger of reactivity. We also took field notes during the interview. Both the tape-recording and the field notes were taken to ensure that the data was accurate. There were no refusals throughout any of the interviews. Furthermore, only the respondents and researchers took part in the study during the interview.

### Reflexivity

The principal investigator had no prior social connections to any of the staff members from the selected hospitals, despite conducting all of the interviews. The principal investigator was unfamiliar with the medical imaging consultation practices and challenges at that particular hospital. Furthermore, the educational background of the investigator was different from the area under investigation. All this together could help the investigator not to influence the situation of the study participants. The ‘bracketing’ technique, on the other hand, which entails suspending researchers’ preconceptions about the subject being investigated, identification, and isolation, was used throughout the entire research process. The researcher’s life experiences, values, and cultural beliefs were all kept open, visible, and transparent.

### Data analysis

A professional with a qualification of BSc in applied human nutrition who had experience in data transcription transcribed all the audio-recorded data verbatim and translated it into English. The translated data was cross-checked with the audio-recorded file to guarantee appropriate transcription and translation. The lead investigator examined the translated data to understand the concepts and related interpretations of the data. Participants had the opportunity to comment on the transcripts, and their comments were taken into account. The translated transcriptions were coded using Open Code 4.02 software by the principal investigator (AM) and the research assistant (TH) after re-reviewing the transcribed texts.

An inductive coding was applied to create a set of codes emerged from the data following the main guiding questions such as: Could you tell me the medical imaging service delivery practice? Could you tell me the consultation modalities which are in practice? Could you tell me the challenges you faced when delivering medical imaging service? Could you tell me the mitigation strategies you have used? What do you recommend for the future to improve the medical imaging service delivery?) Codebook was created in a separate Word document file with the code definitions by the principal investigator (AM) (Additional file 3). Line-by-line coding was employed to detect related patterns by (AM) and (TH). Then, to find themes in the data, codes with similar patterns were combined. Throughout the analysis, many concepts emerged, and these concepts were used to explain comparable phenomena and generate new explanations. The themes’ contributions to comprehending the data were then investigated. Finally, we used an inductive thematic analysis approach to identify major themes that helped to answer the research question by the principal investigator. The work was read and approved by all authors. Moreover, the principal investigator used the COREQ checklist [41] to report the qualitative findings (Additional file 4).

### Theme emergence and development

All tape-recorded data interviews were transcribed verbatim into Amharic (the local language) and then translated into English. The translated transcriptions were imported into OpenCode 4.02 for coding. The definitions for the codebook (Additional file 3) were then created in a separate Word document file. To understand patterns of meaning from data on lived experiences, an inductive approach or data-driven coding was used throughout the data analysis process. Themes were then formed based on the ideas that emerged from the major guiding questions. The relevant quotes were cross-checked with the study’s themes by the investigators. A description and interpretation of the themes’ meanings were used to report the findings. Direct quotes from participants were also used in the report to provide readers with a clear picture of the findings.

### Trustworthiness

To establish the credibility of our analysis, we verified the interview guides by domain experts to ensure the quality of the data. Moreover, the interview guide was pilot tested to check the flow of the questions. The interviews were done in Amharic (the local language of the study area) and translated into English for analysis and interpretation. To achieve sensitivity, verbatim quotes were used in the study to directly report the participant’s voice. Every effort has been made to correctly define the steps of this research method for the sake of transparency, coherence, and replicability. The study applied mixed methods (KII and IDI) to collect the data. The interviews were guided by open-ended questions (Additional file 1 and 2). We used the consolidated criteria for reporting qualitative research (COREQ) guideline to ensure the results’ accuracy and validity. The researchers observed and engaged with research participants for a prolonged period of time.

### Ethical consideration

The University of Gondar Institutional Review Board approved and gave ethical approval for this work (reference no: VP/RTT/05/2554/2021). In addition, supportive letter was also obtained from Amhara Public Health Institute. Throughout the data collection process, all participants provided informed consent, and the study was carried out in accordance with the Helsinki Declaration. Informed written consent was taken from each participant. Furthermore, no personal information provided by participants was used during data analysis, interpretation, or presentation.

## Findings

### Background information of the of study participants

This study interviewed a total of 21 participants (17 KIIs and 4 IDIs). The mean age of the key informants was 34.2 years old (range 27 to 55 years). Similarly, the mean age of the in-depth interviewees was 28 years (range 25 to 32 years). The key-informants’ working experience ranged from 2 to 30 years. All except one of the key-informants had a first degree or higher in terms of their educational background. All the key-informants had an administrative position [Table 1].

**Table 1:** Socio-demographic characteristics of participants, 2021

Interviewee Code	Age	Sex	Educational status	Profession	Current position	Experience (in Year)
KII-1	32	M	Degree	Health Officer	Hospital manager	10
KII-2	30	M	Degree	Nurse	Hospital manager	7
KII-3	40	M	MD^+^	General Surgeon	Hospital manager - delegate	15
KII-4	35	M	MD^+^	Radiologist	Head of radiology department	6
KII-5	55	M	Degree	Radio-technologist	Coordinator of the imaging unit	30
KII-6	30	M	Degree	Radio-technologist	Coordinator of the imaging unit	6
KII-7	38	M	MD^+^	Radiologist	Head of radiology department	8
KII-8	28	M	Diploma	Radiographer	Coordinator of the imaging unit	4
KII-9	34	M	MD	General practitioners	Medical director	4
KII-10	30	M	MD	General practitioners	Medical director	4
KII-11	38	M	Degree	Health Officer	Hospital manager	15
KII-12	31	M	MD	General practitioners	Medical director	2
KII-13	34	M	MD	General practitioners	Medical director	4
KII-14	35	M	MD^+^	General Surgeon	Head of medical department	10
KII − 15	35	M	MD^+^	Internist	Head of internal medicine department	9
KII-16	30	M	MD	General practitioners	Medical director	2
KII-17	27	M	MD	General practitioners	Medical director	2
IDI-18	28	M	6th grade completed	NA	Patient	NA
IDI-19	32	M	Able to read and write	NA	Patient	NA
IDI-2	27	M	Unable to read and write	NA	Patient	NA
IDI-21	25	F	Unable to read and write	NA	Patient	NA

*KII = Key-Informant Interview; IDI = Indepth Interview; MD = Medical Doctor; MD + = Medical doctor with specialty; BSc = Bachelor of Science; NA = Not applicable*

**Fig. 1:**
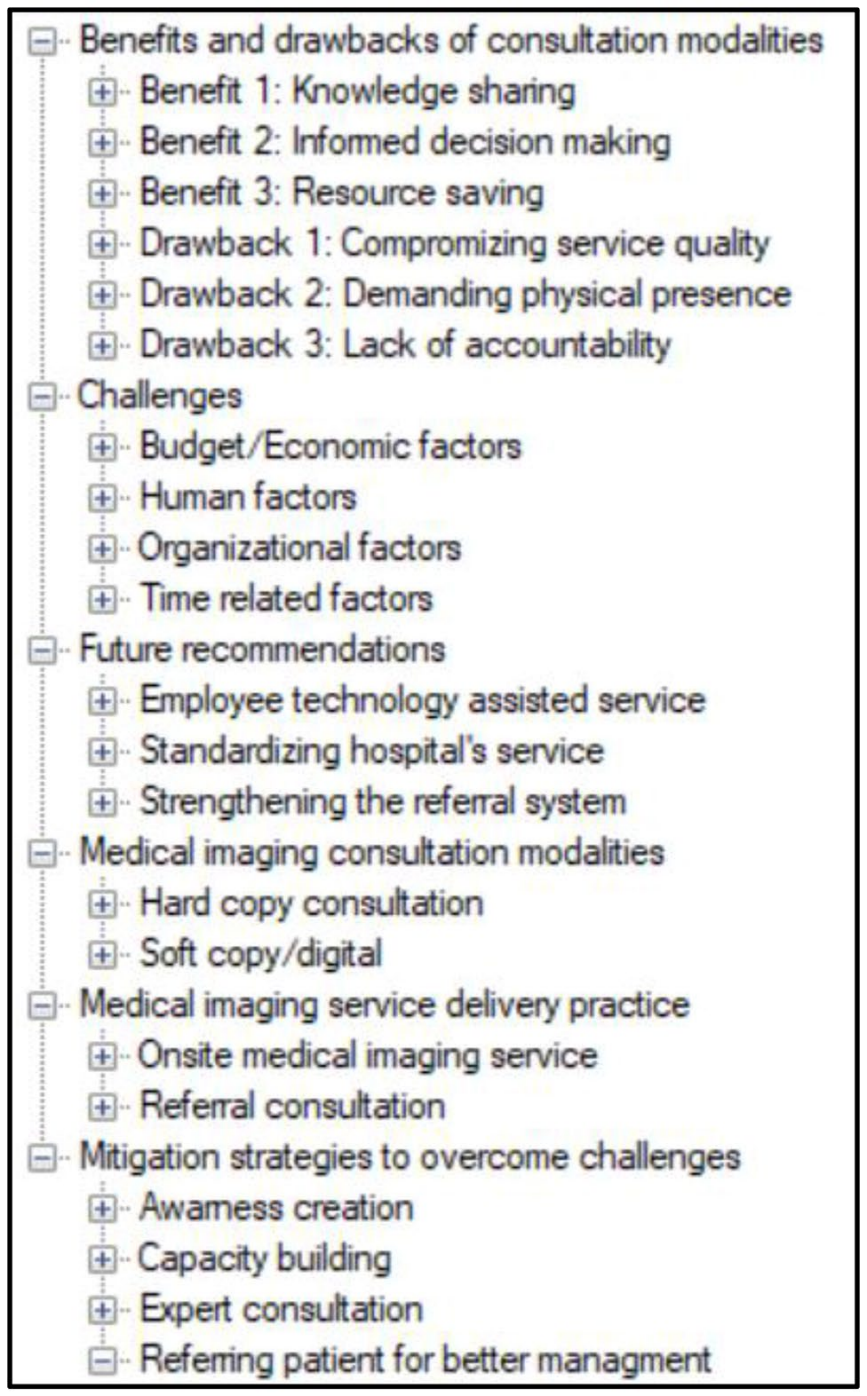
Open code synthesis tree diagram depicting themes and sub-themes of medical imaging practices and their challenges

Following the inductive thematic analysis, six main themes emerged from the data, as shown in Fig. 1. The open code synthesis tree highlights the emerging themes about the practices and challenges of medical imaging consultation at public hospitals in the Amhara region, Ethiopia.

### Theme 1: medical imaging service delivery practices

We found that the most common practices applied during medical image consultations were onsite and referral. Referring clinicians sought referral for image consultation only when onsite radiologists were not available and they were unable to make the image interpretation by themselves. On such occasions, patients are referred either to private imaging clinics or public referral hospitals.

This was explained by the following quote:

*Patients were referred to referral hospitals when physicians perceived that they required additional X-ray or ultrasound investigations. Otherwise, the image interpretation service will be done by the onsite physicians (Hospital manager, 7 years working experience).*

Moreover, when there was a problem related to the imaging equipment or when referring clinicians thought the patient ought to see more senior professionals for optimal care, they occasionally sent the patient to referral hospitals or private imaging clinics.

According to one of the key-informants, failure to provide an onsite consultation service put significant constraints in timely diagnosis and treatment of patients. Furthermore, the lack of onsite services meant patients had to travel long distances looking for medical imaging consultation service. It also means extra expenses and delayed service access. Delay in service and hence diagnosis resulted in disease complications or sometimes death. The informant also added that, in order to avert such scenarios, referring hospitals occasionally invited radiologists for on-site medical imaging services in the form of a short-term paid contract.

A hospital manager reported

*We are currently considering other options to bring radiologists from the referral or teaching university hospitals to provide ultrasound and X–ray onsite services, even if the financial system is challenging. (Hospital manager, 15 years of working experience).*

### Theme 2: medical imaging consultation modalities

The findings indicate that the referring hospital clinicians mostly used either hard copy (printed films) or softcopy/digital image modalities (such as CDs, smart phones, and telegram channels) to send the image to radiologists for consultation.

*In our hospital, CDs and films are the most frequently used options that we are currently using to access experts’ assistance (consultation). In both cases at the time of referral, we remind patients to come back after receiving their radiology report. (Hospital manager, 10 years of working experience).*

### Theme 3: benefits and drawbacks of the consultation modalities

A radiologist key-informant remarked that having access to the digital image consultation made examining the patient much easier. The telegram consultation option allowed patients to save on costs related to accommodation and transportation because it was a free consultation service at a distance provided by the social relationship between the referring clinicians and the radiologist. Furthermore, digital image consultations allowed for optimal image interpretation while maintaining image quality.

*In my opinion, the telegram service is useful to the patient if the patient is not required to be physically there. The majority of today’s machines are digital and can be connected to other digital machines like computers, which supports the transfer of images from one location to another whenever there is a connection. (Radiologist, 8 years of working experience).*

Another hospital manager key informant was also added

*A telegram is a wonderful way to save costs associated with the patient himself and the caregivers for transportation, bed, and food. It will also avoid a car accident, which might probably occur during transportation. (Hospital manager, 15 years of working experience).*

Digital image consultation simplified service delivery for radiologists by allowing them to zoom in and zoom out to see the image from different angles. Moreover, the digital image preserves the image’s quality and protects the patient from misdiagnosis and treatment.

*Telegram and CD consultations are normal in terms of maintaining quality and enabling us to see the image by zooming in and out. (Radiologist, 6 years of working experience).*

Both hardcopy and softcopy/digital image consultation modalities were viewed as advantageous for consulting clinicians as means for exchanging and sharing professionals’ expertise. Accessing expert knowledge could help referring clinicians to improve clinical decisions making and possibly minimize additional referrals in the same scenario.

*As we have a group discussion, we can update ourselves on the clinical report we obtained. It is an important approach to self-update, which implies a consulting medical doctor can share knowledge with senior radiologists. (Medical director, 4 years working experience).*

Hardcopy/film-printed consultation modalities have several drawbacks compared to softcopy/digital image consultation modalities. Of these, low film quality, vulnerability to damage due to poor handling, and a higher rate of repeat-studies which could expose patients to unnecessary radiation exposure were some of the challenges. A key informant reported

Both the CD and film have no difference in usage, but when we use films, there is a chance of being blurred and does have repeated taking of images, putting patients at risk of radiation exposure. (Medical director, 2 years of working experience).

Another key-informant also mentioned that incomplete information on requested studies was is a challenge during the referral consultation. As a result of lack of patient access, radiologists were often challenged to produce accurate imaging reports. The radiologist is unable to obtain relevant clinical information directly from the patient because X-ray images are frequently submitted to radiologists for consultation by caretakers or family members. Due to this, incomplete information may result in incorrect interpretation, which may affect patient diagnosis and treatment.

We can’t get sufficient information, such as physical examination details and patients’ previous history, when the patient image is sent to us by caregivers or other individuals. On such occasions, we have tried to access information from the requesting clinician via his cell phone because it is difficult to determine how long the patient has been with the disease. (Radiologist, 6 years working experience).

On the other hand, even if the telegram consultation option was a free and volunteer-based service, a radiologist’s lack of commitment was one of the major challenges that could cause a delayed response. Furthermore, ensuring accountability for the delay was impractical because there was no legal institutional agreement between the referring and referral hospitals. Overall, a delayed radiology report may result in delayed diagnosis and treatment. This idea was supported by a quote from a key informant report:

*Because we do not have a contract agreement with radiologists, we are having difficulty using telegram. The radiologists are not fully responsible for our consultation because the service is volunteer-based. They will not be held accountable. They may also overlook the request and sometimes fail to respond at all. (Medical director, 4 years of working experience).*

Participants had varying preferences for consultation methods for medical imaging. However, their justification for favoring or rejecting it appeared plausible. One of the key informants reported:

*I prefer both telegrams and CDs instead of image films because the rejection rate for films is very high due to the poor image quality. However, in telegram consultation, there is also an issue of accountability during the occurrence of medico-legal issues since it is a free, volunteer-based service. (Radiologist, 8 years working experience).*

Another key informant, on the other hand, claimed that telegram consultation was much better than CD consultation.

*I preferred telegram for X-ray, CT scan, and MRI images to access the radiologists remotely without letting patients travel further. On the other hand, CDs are very prone to damage when they encounter scratches because of poor handling. (Radiographer, 4 years of working experience).*

### Theme 4: Challenges

The influencing determinants for successful medical imaging service delivery reported by the participants were summarized under four subthemes; namely: (a) organizational; (b) time-related; (c) human; and (d) financial/economical.

### Subtheme A: organizational determinants

Providing medical imaging services to consulting hospitals was especially challenging due to several organizational constraints. Most of the healthcare services delivery delays were caused by organizational determinants. Various administrative reasons contributed to the delay. Referral hospitals lacked a system to provide imaging consultation services for those images sent from the referring facilities unless the patient had gone through the standard referral pathway for clinical service. Moreover, frequent power outages, delayed equipment maintenance and servicing, a shortage of imaging films, and lack of trainings were all significant organizational obstacles. One of the hospital managers reported:

*Oh! Quality healthcare service delivery is a critical challenge, specifically for primary hospitals. We have been without an X-ray machine for the last 4 years because of a lack of maintenance. In addition, due to the strictness of the hospitals’ rules and regulations, radiologists working at the referral hospitals are not accepting consultation requests from primary hospitals. (Hospital manager, 7 years of working experience).*

The informant also added:

*As a result, referred patients for the X-ray image consultation service are not accessing the X-ray image consultation based on the given request. Surprisingly, the patient is asked to start from scratch and get an imaging request again from that hospital. (Hospital manager, 7 years of working experience).*

On the other hand, despite the fact that capacity-building is crucial for improving the performance of health professionals, on-site and off-site trainings were inadequate. Frequent X-ray machine failures could be an indication that imaging technicians lack the necessary manipulation skills.

*Staff members of the department are not getting adequate technical training on how to use the imaging machines properly. We are operating the machines by familiarizing ourselves by reading the manuals and the awareness created by the equipment provider’s technical personnel during the machine deployment phase. (Radiographer, 4 years of working experience).*

The key-informant also added that imaging machine malfunctions are uncommon due to the lack of technical knowledge of imaging technicians.

*Surprisingly, there are scenarios where machines are still out of service due to improper operation. (Radiographer, 4 years of working experience).*

### Subtheme B: Time related determinants

The study area referral hospitals had a high patient load and a limited number of radiologists, making timely consultation services difficult. As a result, accessing the medical imaging consultation service without a long appointment time was challenging.

*I have been here for the last six days without getting any service, even though I was given an appointment. I have been wasting three days without contacting the requesting physician. (28-years-old, male patient).*

Patients preferred private imaging clinics even though the service costs were significantly higher than those of government health facilities. This is because private imaging clinics provide faster service than government hospitals.

*I prefer private imaging clinics considering the timely service, even though the service cost is very expensive. (25-year-old female patient).*

### Subtheme C: Human determinants 

The provision of radiologic services was severely hindered by the paucity of radiologists and technological expertise. Due to this, patients preferred private healthcare clinics.

In the standard, it is stated that one professional should give services up to 15 patients per day. However, we have been providing ultrasound services for up to 40 patients per day. Surprisingly, due to the high volume of patient flow, there are cases where we are forced to give appointments for 2–3 days. (Radio-technologist, 30 years of working experience).

However, due to the lack of imaging technicians with the necessary maintenance skills, imaging equipment lasts a long time without providing service. Due to the dearth of radiographers with the necessary capabilities, hospitals invite technicians from other sectors. Moreover, their willingness to timely fix imaging equipment was a challenge for them. All these together interrupted the medical imaging service provision frequently.

We invited the imaging technicians by covering all the associated costs; however, they did not come as per the agreed schedule. Some of the reasons for their delay were that the location is remote (inaccessible to transportation) and the issue of security. (Hospital manager, 10 years of working experience).

Moreover, patients’ lack of trust in some of the available options for image consultation had an impact on their satisfaction with the imaging service they received.

A hospital manager key-informant stated that

*Some rural and uneducated patients believed that they didn’t get the imaging service unless they were given a hard copy (film prints). (Hospital manager, 10 years of working experience).*

### Subtheme D: Budget/Economic determinants

According to key-informants, there was a scarcity of imaging films on the market, in addition to significant financial constraints, making it difficult for hospitals to provide the service on time.

*We are unable to purchase an adequate number of films due to a lack of funding and the high cost of image films. In addition to the financial constraints, it is challenging to access a sufficient number of image films on the market. (Radio-technologist, 6 years of working experience).*

Patients had to travel a great distance to use radiologists’ image interpretation service due to the lack of the service at their nearby hospital, which forced them to spend extra money for accommodation and transportation.

*The cost associated with the imaging service is unaffordable for me. I have been here along with my two caregivers for the last six days, wasting a lot of money since most of the advanced imaging services were not accessible within a short period of time. For example, we spent nearly 13, 000 Ethiopian Birr for imaging services (ultrasound and CT scan from private clinics) (28-year-old male patient).*

Another interviewee also added that the cost of food, bed rent, and transportation was very expensive.

*You may be aware that transportation, food, and bed-rent are all becoming increasingly expensive. For example, I paid 200 Ethiopian Birr for a single day’s bed rent, which was very costly for me. In general, if the services were available at our nearby hospital, we could save the costs associated with transportation, food, and bed rent. (27-years-old, male patient).*

### Theme 5: Mitigation strategies to overcome challenges

The following alternatives were taken by consulting clinicians to address problems they experienced throughout the image consultation process. Among those, referring patients to better management was the most frequently used option practiced by the consulting clinicians. In most cases, they refer patients when they encounter failure of a medical imaging machine, a shortage of imaging films, or when the patient has complications. Moreover, when clinicians planned the patient’s demand for senior specialists, the patient was not requested to give an image there to protect him from unnecessary radiation.

*If a patient is referred for better management, the patient has the chance to be seen there by senior physicians. The recommended image requested by the consulting physician may be cancelled or more extra imaging services may be added (for example, echocardiography and ultrasound in addition to X-ray). (Radiographer, 4 years of working experience).*

Furthermore, participants reported the strategies they used to overcome challenges while providing medical imaging services. Some of the mitigation strategies were the availability of short-term and long-term training opportunities, reminding patients about the benefits and drawbacks of image consultation modalities, making patients aware of the consequences of a consultation delay, and communicating with radiologists via cell phones.

*We requested the health bureau on how to improve the academic status of our professionals through long-term training and short-term training. We have a plan to capacitate our physicians by at least facilitating two days of training, inviting radiologists from teaching universities. (Hospital Manager, 7 years of work experience).*

### Theme 6: Future recommendations

Participants offered a number of suggestions to enhance the imaging services. Most of the participants reported that the best course of action to enhance the medical imaging consultation would be to assist the service with technology by upgrading the current infrastructure and allocating an adequate budget. They stated that patients were compelled to travel long distances to referral hospitals looking for image interpretation service. To minimize challenges related to further travel, participants recommended the medical consultation service be assisted by technology. Assisting the service with teleradiology would improve the service quality by decreasing the radiology report time as well as eliminating the cost associated with further travel.

*There should be a shift from the traditional way to technology-based. If we implement teleradiology, life will be easy for both professionals and patients as it solves many problems, especially those related to distance. (Radiologist, 8 years of working experience).*

Another in-depth interview participant stated,

*In my opinion, it would be very good if the service were available at our nearby hospital. This is because if we could access the service at our nearby hospital, it would save us time and money. In addition, although we cost too much, the service should be given on time. (28-year-old male patient).*

Participants reported that consulting hospitals were inviting radiologists to offer on-site services in an effort to minimize the obstacles related to distance. However, due to a lack of funding, they found it difficult to continue with their plan., Participants recommended that the government would allocate an adequate budget to deliver the medical imaging service in a sustainable manner.

*We also tried to invite radiologists to come to our hospital and deliver the service once or twice a week by covering all the service, transportation, and accommodation costs, but due to the financial constraints, we were unable to continue. But I wish there would be adequate budget allocation. (Medical director, 2 years of working experience).*

The key-informants recommended standardizing imaging facilities and availing of extra hard drives to keep images for a long time. They also added that the computer’s hard disk wouldn’t be large enough to store all the images due to the high volume of patient flow. Additionally, they also suggested that having adequate infrastructure, reducing unnecessary prescriptions, strengthening the referral system, improving the response times, creating opportunities for capacity building, and rewarding experts were equally crucial.

*Unnecessary prescriptions should be minimized because they can cause unnecessary delays for other patients. For example, if a person needs surgery, it doesn’t matter if the ultrasound or X-ray results are positive; it is a waste of time. When you minimize such instances, you are bringing deserving patients to the front. (Medical director, 15 years of working experience).*

Similarly, expanding the existing infrastructure and availing the imaging equipment were critical for improving the delivery of medical imaging services. For example, though teleradiology is a cross-cutting solution to address issues related to distance, it would be impractical without the hospital’s information communication and technology infrastructure facilities.

*Another option is to add imaging machines and units to increase the number of patients to be treated per day. In our case, for example, even if we have additional radiologists, we are not utilizing them effectively because we do have only one machine and one room. (Medical director, 15 years of working experience).*

Furthermore, attention should be given to unfair image requests because such requests could consume other eligible patients’ time.

*Even though we tried to make them aware of this several times, they didn’t understand us. We believe that they are doing it again and again to confirm our clinical report with their diagnosis. But we recommend that they be informed about the importance of avoiding unnecessary image requests. (Radiologist, 6 years of working experience).*

## Discussion

The purpose of this study was to explore image consultation practices and challenges from the perspectives of health professionals and patients in the Amhara Regional State Public Hospitals. We found that all participants acknowledged the problem of conventional medical imaging consultation. Suggested measures included strengthening the referral system by assisting with technology, minimizing unnecessary referral requests, and enhancing the skills of professionals through short-term and long-term capacity-building training.

This finding is supported by a review study conducted in Africa [14]. The review reports that limited human resources, insufficient facilities and opportunities for education and training, and a low level of integration of imaging into the entire health service delivery system were the major challenges in the practice of medical imaging service delivery. The authors of this study confirmed that onsite consultations by inviting radiologists to the referring hospitals and referral consultations by sending the images to referral hospitals or private imaging clinics were the two most common approaches in practice. Alternatively, for better management by senior professionals, consulting clinicians physically refer patients to referral hospitals due to the paucity of radiologists in Ethiopia. According to a retrospective study conducted in Ethiopia, from 1990 to 2021, 430 students graduated in the field of radiology at five universities [42]. The figure demonstrates the severity of the radiology professional shortage.

Hardcopy and softcopy (digital image) consultation modalities were frequently used during the referral consultation process. Printed-film was the only option for hardcopy image consultation, whereas CDs and social media Telegrams were used for softcopy consultation. However, previous studies indicated that, the most popular social media platforms for radiologists to communicate radiological information were WhatsApp and Facebook [43–45]. The variation could be due to participants’ previous exposure and the functionalities of the social media. For example, in Telegram, users can upload large size files; it is easy to sign up and use compared to other social media options [46]. In addition, Telegram is the most popular platform for communication by employees at the workplace in the context of Ethiopia [47].

The benefits and drawbacks of the hardcopy and digital image consultation procedures were reasonably expressed by the participants. The key challenges experienced by the majority of the key informants during the medical imaging consultation practice were poor image quality and slow response times. Poor image quality might result from improper handling of image transportation options, as they are susceptible to damage by dust, scratches, and unnecessary folding. This implies that poor image quality increases the rejection rate, subjecting patients to unnecessary radiation and deterring them from receiving timely medical care [48].

Similarly, inadequate imaging technician skills could also contribute to poor image quality which in turn influences the radiologist to interpret it accurately. Previous studies revealed that inadequate resolution, imaging plate artifacts, and a lack of post-processing functions were the main drawbacks of screen film radiography [1, 49]. According to our findings, participants experienced sometimes long response time to receive radiology reports during the telegram consultation process due to lack of radiologists commitment. This is because the telegram consultation was a free and voluntary service that was done through informal communication between the referring clinician and the radiologist. As a result, the radiologist commented and returned the radiological report to the consulting physicians when he or she had free time.

Although the referral approach was the most popular approach to image consultation, the incompleteness of the referral information had a substantial impact on the provision of medical imaging services. Our finding is consistent with previous findings done elsewhere [50–52]. This implied that when radiologists received detailed information about the patient during the consultation process, they could generate a more credible and trustworthy radiological report that can safeguard the patients from misdiagnosis and treatment. Previous research work confirmed that the acts of radiographers in completing missing data during radiology referrals enhance the provision of high-quality medical services [32]. However, in some of the referral consultations, radiologists were unable to access the patient to obtain the information they missed because the patient’s family or caregivers were responsible for transporting the image during the consultation process [50, 53]. If a substantial portion of referrals for imaging tests were improperly prepared, it would result in an erroneous investigation [54].

The availability of adequate resources was considered as a cross-cutting solution for challenges related to medical imaging service provision [5, 55]. However, key-informants in this study confirmed that providing medical imaging services was difficult due to a shortage of radiologists, inadequate imaging equipment, and poor infrastructure such as a local area network and electricity. Additionally, they also noted that frequent interruptions of imaging equipment and a lack of timely maintenance and service were all critical issues preventing clinicians from offering high-quality healthcare services.

According to previous research, a shortage of technical experts, insufficient infrastructure, and a lack of professionals are still common challenges for LMICs [3, 9, 43, 55]. Participants indicated that, imaging machine interruption was largely caused by technicians’ technical inability. It was pointed out that most of the digital imaging machines were obtained by donation, and the majority of radiologists agreed that the drawbacks of donated equipment outweighed the cost savings [56]. In response to challenges with medical equipment failure, the Federal Ministry of Health (FMOH) of Ethiopia designed a plan and included a strategy called *“establish medical equipment maintenance centers and increase their capacity and functionality”* under Health Sector Transformation Plan II (HSTP II) [27, 55].

Findings showed that only 30% of the radiographic images could be correctly interpreted by physicians and students due to a lack of adequate knowledge [57]. As a solution, enhancing the skills of consulting clinicians working at distant hospitals through short-term training is recommended [32, 57–59]. To further reduce medical errors, it is advised that quick consultation of medical images in quest of expert judgment is essential [60, 61].

This study demonstrated that, frequent power outages, shortages of consumables, and bureaucratic procurement processes were other contributing determinants for the delay of medical imaging service delivery. Previous study findings revealed that a shortage of consumables contributed to the majority of the medical imaging service delays [30, 43]. In this study, the following mitigating strategies, such as making appointments, referring patients for better management, and inviting a radiologist to provide onsite medical imaging services, were practiced as solutions to improve the service provision by the consulting clinicians at the referring hospitals. This finding is supported by other studies stating the need for more training of consultant radiologists and radiographers by higher academics to address the shortage and improve access to medical imaging services [2].

Our study revealed that medical imaging consultation should be assisted with technology such as teleradiology in order to optimally utilize the limited number of specialists. According to prior research, referrers (consulting clinicians) reported that the virtual consultation approach was extremely helpful when practicing conventional consultation was challenging because of location or time [62]. Another study also documented that digital technology still improves patient outcomes and makes healthcare safer even though it lacks the accurate data that is generally gathered during a physical assessment [63]. Technology-assisted consultation using teleradiology, for instance, has a considerable impact on patients’ diagnosis and treatment times, health outcomes, and satisfaction [43, 64] by decreasing the need for face-to-face presence without compromising the patient’s perception of the quality of care [65].

Ethiopia’s federal ministry of health devised short-term (1–3 years), medium-term (3–5 years), and long-term (5–10 years) plans to improve healthcare service delivery by assisting with technology [28, 66]. One of these was the recommendation to introduce teleradiology in public hospitals as a short-term strategy under pillar II [28, 66]. However, the absence of adequate ICT infrastructure is still a challenge in making services available [66]. Other suggestions made by participants for future medical imaging service improvements included strengthening the referral system, reducing unnecessary image requests, allocating an adequate budget, incentivizing radiologists, and capacitating radiographers.

### Strengths and limitations of the study

This is the first study of its kind in Ethiopia to shed light on how medical imaging consultation is practiced and what challenges are influencing the practice. The study enlisted a diverse group of key-informants to investigate the practice and its associated challenges. Though the study had some strength, it also had some limitations: (1) The findings of the study can only be applied to this specific research area because the sample size obtained was not representative of the overall population; (2) Participants may respond to suggested responses because self-reported data is susceptible to social desirability bias; (3) We also acknowledged that the concept of data saturation is debatable and that new themes may have emerged from additional interviews [67] and (4) Patient participants may produce fewer insights regarding the challenges of providing medical imaging services than key informants due to their limited exposure to the hospital’s service delivery practice.

### Implication for the practice

The study findings suggested that patients are still suffering from unintended transportation and accommodation expenses by travelling long distances to access radiologists’ services. The findings could also suggest that, in most cases referring physicians were not in a position to offer timely diagnosis and treatment for their patients due to the radiology report delay. The medical radiology report delay could eventually compromise healthcare service delivery. These all together imply that adopting teleradiology could be the best solution to avail virtual consultation that could help to reduce the radiology report response time.

## Conclusions

This research explored six themes that emerged from medical imaging consultation practices and their associated challenges. The two practices onsite and referral were common to deliver the medical imaging service. The referring clinicians used both film print and softcopy (digital image) consultation modalities during the referral consultation practice, despite each of them had its own drawbacks. The main obstacles affecting the medical imaging consultation practice were organizational, financial, time, and manpower-related. These issues could have a considerable impact on the referring clinicians’ ability to deliver timely diagnosis and treatment services. To overcome these challenges, certain components would be recommended as key strategies to improve the consultation practice, such as strengthening the referral system, assisting the service with technology (specifically teleradiology), and availing of short-term and long-term capacity building activities.

## Electronic supplementary material

Below is the link to the electronic supplementary material.

Additional file 1. KIIs interview guide.

Additional file 2. IDIs interview guide.

Additional file 3. Codebook.

Additional file 4. COREQ guideline.

## Data Availability

The datasets used and/or analyzed during the current study are available upon reasonable request from the corresponding author.

## List of Abbreviations

CD: Compact Disk
CI: Confidence Interval
CT: Computed Tomography
FMOH: Federal Ministry of Health
HSTP: Health Sector Transformation Plan
ICT: Information Communication Technology
LMICs: Lower and Middle Income Counties
mHealth: Mobile Health
MRI: Magnetic Resonance Imaging
SSA: Sub-Saharan Africa
WHO: World Health Organization
