# Supplementary material for: Medical imaging consultation practices and challenges at public hospitals in the Amhara regional state, Northwest Ethiopia: a descriptive phenomenological study
Source: BMC Health Serv Res. 2023 Jul 24;23:787. doi: 10.1186/s12913-023-09652-9 (PMC10367423; doi:10.1186/s12913-023-09652-9)
Supplement: Supplementary file 1 — Additional file 1. KIIs interview guide. [file 12913_2023_9652_MOESM1_ESM.docx]

**Key Informant Interview Questionnaire Guide (English Version)**

Medical Imaging Consultation Practices and Challenges at Public Hospitals in Amhara Regional State, Northwest Ethiopia: A Qualitative Study

**An information sheet used to ask permission**

**Information sheet:** Read the statements to the respondent

**Purpose of the research project:** Dear Participants, the University of Gondar Institute of Public Health and Principal Investigator (Araya Mesfin) are conducting the study Central Gondar and South Gondar zones among selected public hospitals, Northwest Ethiopia. This study is ***aimed to explore medical imaging service consultation practices and their challenges***. Hence, you have been chosen to participate in this study.

**Procedure:** in order to collect our data, we invite you to take part in our project. If you are willing, you need to understand and sign on the consent form. We will ask some questions.

**Risks and/or discomforts of being in this study:** There are no a reasonably foreseeable (expected) risks of this study except time consumptions.

**Benefits/ payments:** If you participate in this research project, the study's findings will have both immediate and indirect benefits for you, your family, and the community as a whole, who will get services in the future.

**Confidentiality:** We will keep the confidentiality of the information that we collect from you. The record of this study will be kept confidential. Research records will be kept in a locked file, and all the electronic information will be coded and secured using password security. We will not include any information in any report we may publish that would make it possible to identify you.

**Right to Refuse or withdraw:** The decision to participate in this study is entirely up to you. You may refuse to take part in the study at any time without affecting your relationship with the investigator of this study. You have the right not to answer any single question, as well as to withdraw completely from the interview at any point during the process.

**Person to contact:** If you want to know more information you can contact; Mr. Araya Mesfin
by Tel: mobile +251-918713813. If you agree to take part in the above-mentioned study, please sign below and write the date (in the presence of other family members). Please answer all of the questions in the booklet. Instructions for responding to the booklet's several questions are provided at the top of each page.

**Consent Form**

I, the undersigned, have understood the objective of the project aimed to “explore medical imaging service consultation practices and their challenges at Central Gondar South Gondar zones among selected public hospitals, Northwest Ethiopia”, and agreed to be included in the study as explained by the researchers.

I decided to take part in this in-depth interview. I also agree to the use of anonymous quotes and realize that they will not be traceable back to me. With due understanding of the aforementioned information, are you willing to participate in the study?

I______________, the member of the research team would like to inform you that the purpose of this interview is to explore medical imaging service consultation practices and their challenges. Your accurate responses are vital for the quality of our research outputs. Your responses are confidential. By participating in this research there is no potential harm/benefit.

Are you willing to participate: Yes_______ No______
If you are willing to participate Please put your Signature: ______________.
No (Terminate the interview)

Interviewer Name ___________Signature ________ date _______

**Key Informant Interview Guide Questions for Radiologists**

| **Basic socio-demographic characteristics** | |
| --- | --- |
| Interviewer _______________________ | Interviewee code : _________________  Age: ______________  Sex: _______  Educational Level: ______________  Work experience (in years):_______________ |
| Date: dd/mm/yy: ---------/-------/------ | Have you agreed for the interview?   1. Yes 2. No |
| Beginning time of the interview: **-----: -----** | End time of the interview: **----: ----** |
| Recorder ID: _______________ | Recording # _______________________ |

| **Introduction** | |
| --- | --- |
| We are gathering today because we are interested in learning more about concerns regarding radiology service provision. We are specifically interested in learning about concerns that patients related to the radiology image consultation practices and their challenges. We are going to talk about each of these topics.  To make you more comfortable, you do not need to share what you experience specifically. You can react what you know may be concerns for other patients like you. | |
| **Opening: Ice-Breaker** | |
| To begin would you like to tell me a little bit about yourself? You are not required to tell your name. Please wander around and share your favorite aspect of the day or anything else you like. | |
| **Detail question guide for key informant in-depth interview ( Radiologists)** | |
| **Questions** | **Probing questions** |
| 1. Could you tell us the radiology service delivery practice in your organization? |  |
| 1. Currently, do you provide referral radiology image consultation service? | - If not, could you explain the reason? - How do you entertain referral consultation case? |
| 1. What are the medical image consultation options which are have been practiced? | - Which image consultation modality you are supporting? And why? |
| 1. How do you see patients waiting time to get radiology service? | - Could you explain some contributing factors for the delay? |
| 1. Where is your preference image consultation facility? | - Private imaging clinics, government referral hospitals? - Describe the reasons why you prefer them? |
| 1. Could you describe some drawbacks associated with the current practice in order to get image interpretation service? | - Could you explain the advantages and disadvantages? - Explain in terms of image quality, time, accessibility |
| 1. How do you see the contribution of current practice for end users (patients)? | - Explain it in terms of ensuring appropriate imaging interpretation service? - Explain it in terms of waiting time and cost? |
| 1. How do you describe the image interpretation practice? (current Vs. previous service provision) | - Explain in terms of workload, working hours |
| 1. What strategy do you recommend for the future to improve the radiology referral service? | - Could you explain some possible suggestions which will benefit the referring physicians, patient and you as well? |

Thank you for answering all our questions!!!

Maybe you have thought of something that we have left out. Is there anything else that you would like to tell me about your experience regarding institutional delivery?

**Key Informant Interview Guide Questions for Consulting Clinicians**

| **Basic socio-demographic characteristics** | |
| --- | --- |
| Interviewer _______________________ | - Interviewee code : _________________ - Age: ______________ - Sex: _______ - Educational Level: ______________ - Work experience (in years):______________ |
| Date: dd/mm/yy: ---------/-------/------ | Have you agreed for the interview?   1. Yes 2. No |
| Beginning time of the interview: **-----: -----** | End time of the interview: **----: ----** |
| Recorder ID: _______________ | Recording # _______________________ |

| **Introduction** | |
| --- | --- |
| We are gathering today because we are interested in learning more about concerns regarding radiology service provision. We are specifically interested in learning about concerns that patients related to the radiology image consultation practices and their challenges. We are going to talk about each of these topics.  To make you more comfortable, you do not need to share what you experience specifically. You can react what you know may be concerns for other patients like you. | |
| **Opening: Ice-Breaker** | |
| To begin would you like to tell me a little bit about yourself? You are not required to tell your name. Please wander around and share your favorite aspect of the day or anything else you like. | |
| **Detail question guide for key informant in-depth interview ( Radiologists)** | |
| **Questions** | **Probing questions** |
| 1. Could you tell us the radiology service delivery practice in your organization? |  |
| 1. What are the medical image consultation options which are have been practiced? | - Which image consultation modality you are supporting? And why? |
| 1. How do you evaluate the contribution of current practice form end-users (patients) perspective? | - Explain it in terms of ensuring appropriate imaging interpretation service - Explain it in terms of waiting time, accessibility, cost, service quality, providing timely treatment - Explain it in terms of updating knowledge |
| 1. Could you describe some drawbacks associated with the current radiology image consultation practice? | - Explain in terms of image quality, time, accessibility |
| 1. How do you see patients waiting time to get radiology service? | - Could you explain some contributing factors for the delay? |
| 1. Have you received feed-backs from radiologists during the consultation process? | - Explain it in terms of appropriate service provision - Explain it in terms of updating your knowledge and its impact on service the healthcare service delivery |
| 1. How do you describe the image interpretation practice? (current vs. previous service provision) | - Explain in terms of workload, working hours |
| 1. What strategy do you recommend for the future to improve the radiology referral service? | - Could you explain some possible suggestions which will benefit the referring physicians, patient and you as well? |

Thank you for answering all our questions!!!

Maybe you have thought of something that we have left out. Is there anything else that you would like to tell me about your experience regarding institutional delivery?

**Key Informant Interview Guide Questions for Hospital Managers**

| **Basic socio-demographic characteristics** | |
| --- | --- |
| Interviewer _______________________ | - Interviewee code : ____________ - Age: ______________ - Sex: _______ - Educational Level: ______________ - Work experience (in years):___________ |
| Date: dd/mm/yy: ---------/-------/------ | Have you agreed for the interview?   1. Yes 2. No |
| Beginning time of the interview: **-----: -----** | End time of the interview: **----: ----** |
| Recorder ID: _______________ | Recording # _______________________ |

| **Introduction** | |
| --- | --- |
| We are gathering today because we are interested in learning more about concerns regarding radiology service provision. We are specifically interested in learning about concerns that patients related to the radiology image consultation practices and their challenges. We are going to talk about each of these topics.  To make you more comfortable, you do not need to share what you experience specifically. You can react what you know may be concerns for other patients like you. | |
| **Opening: Ice-Breaker** | |
| To begin would you like to tell me a little bit about yourself? You are not required to tell your name. Please wander around and share your favorite aspect of the day or anything else you like. | |
| **Detail question guide for key informant in-depth interview ( Radiologists)** | |
| **Questions** | **Probing questions** |
| 1. Could you tell me how radiology service image interpretation is going on in you hospital? |  |
| 1. What are the medical image consultation options which are have been practiced? | - Which image consultation modality you are supporting? And why? |
| 1. How do describe the availability of resources for the current system? | - Explain in terms of availability of image film, resource (budget) allocation from RHB/FMOH - Explain in terms of resources utilization |
| 1. How do you see the availability of appropriate personnel? | - Maintenance personnel/timely maintenance - Probe: If they are not available, how do you get the maintenance service? |
| 1. How do you see patients waiting time to get radiology service? | - Could you explain some contributing factors for the delay? |
| 1. Could you tell us the challenges related to radiology service? | - Explain it in terms of patient satisfaction - Explain it in terms of budget allocation/maintenance - Explain it in terms of availability of radiology professionals |
| 1. Did you encounter clients complain related to the radiology service? | - How do you manage it? |
| 1. What strategy do you recommend for the future to improve the radiology referral service? | - Could you explain some possible suggestions which will benefit the referring physicians, patient and you as well? |

Thank you for answering all our questions!!!

Maybe you have thought of something that we have left out. Is there anything else that you would like to tell me about your experience regarding institutional delivery?
