# Supplementary material for: Medical imaging consultation practices and challenges at public hospitals in the Amhara regional state, Northwest Ethiopia: a descriptive phenomenological study
Source: BMC Health Serv Res. 2023 Jul 24;23:787. doi: 10.1186/s12913-023-09652-9 (PMC10367423; doi:10.1186/s12913-023-09652-9)
Supplement: Supplementary file 2 — Additional file 2. IDIs interview guide. [file 12913_2023_9652_MOESM2_ESM.docx]

**In-depth Interview Questionnaire Guide (English version)**

Medical Imaging Consultation Practices and Challenges: **An information sheet used to ask permission**

**Key informant In-depth interview guide question for patients attending medical imaging service**

**Information sheet:** Read the statements to the respondent

**Purpose of the research project:** Dear Participants, the University of Gondar Institute of Public Health and Principal Investigator (Araya Mesfin) are conducting the study Central Gondar and South Gondar zones among selected public hospitals, Northwest Ethiopia. This study is **aimed to explore medical imaging service consultation practices and their challenges**. Hence, you have been chosen to participate in this study.

**Procedure:** in order to collect our data, we invite you to take part in our project. If you are willing, you need to understand and sign on the consent form. We will ask some questions.

**Risks and/or discomforts of being in this study:** There are no a reasonably foreseeable (expected) risks of this study except time consumptions.

**Benefits/ payments:** If you participate in this research project, the study's findings will have both immediate and indirect benefits for you, your family, and the community as a whole, who will get services in the future.

**Confidentiality:** We will keep the confidentiality of the information that we collect from you. The record of this study will be kept confidential. Research records will be kept in a locked file, and all the electronic information will be coded and secured using password security. We will not include any information in any report we may publish that would make it possible to identify you.

**Right to Refuse or withdraw:** The decision to participate in this study is entirely up to you. You may refuse to take part in the study at any time without affecting your relationship with the investigator of this study. You have the right not to answer any single question, as well as to withdraw completely from the interview at any point during the process.

**Person to contact:** If you want to know more information you can contact; Mr. Araya Mesfin
by Tel: mobile +251-918713813. If you agree to take part in the above-mentioned study, please sign below and write the date (in the presence of other family members). Please answer all of the questions in the booklet. Instructions for responding to the booklet's several questions are provided at the top of each page.

**Consent Form**

I, the undersigned, have understood the objective of the project aimed to “explore medical imaging service consultation practices and their challenges at Central Gondar South Gondar zones among selected public hospitals, Northwest Ethiopia”, and agreed to be included in the study as explained by the researchers.

I decided to take part in this in-depth interview. I also agree to the use of anonymous quotes and realize that they will not be traceable back to me. With due understanding of the aforementioned information, are you willing to participate in the study?

I______________, the member of the research team would like to inform you that the purpose of this interview is to explore medical imaging service consultation practices and their challenges. Your accurate responses are vital for the quality of our research outputs. Your responses are confidential. By participating in this research there is no potential harm/benefit.

Are you willing to participate: Yes_______ No______
If you are willing to participate Please put your Signature: ______________.
No (Terminate the interview)

Interviewer Name ___________Signature ________ date _______

**In-depth interview guide question with patients attending medical imaging service**

| **Basic socio-demographic characteristics** | |
| --- | --- |
| Interviewer _______________________ | - Interviewee code : _________________ - Age: ______________ - Sex: _______ - Educational Level: ______________ - Residence: 1. Urban 2. Rural |
| Date: dd/mm/yy: ---------/-------/------ | Have you agreed for the interview?   1. Yes 2. No |
| Beginning time of the interview: **-----: -----** | End time of the interview: **----: ----** |
| Recorder ID: _______________ | Recording # _______________________ |

| **Introduction** | |
| --- | --- |
| We are gathering today because we are interested in learning more about concerns regarding radiology service provision. We are specifically interested in learning about concerns that patients related to the radiology image consultation practices and their challenges. We are going to talk about each of these topics.  To make you more comfortable, you do not need to share what you experience specifically. You can react what you know may be concerns for other patients like you. | |
| **Opening: Ice-Breaker** | |
| To begin, would like you to tell me a little bit about yourself. You are not required to tell your name. Please wander around and share your favorite aspect of the day or anything else you like. | |
| Detail question guide for in-depth interview ( patients who are attending radiology service) | |
| Questions | Probing questions |
| 1. Have you received comprehensive information about the radiology from the imaging technician concerning the procedure to be performed? | - Pre-treatment preparation and/or instructions; post-treatment and/or discharge instructions; fee information; risks - How do you see the imaging unit dressing room? Was it good to ensure your privacy? |
| 1. How do you get the medical imaging consultation service? | - Have you accessed the service from private or governmental hospital? - Could you tell me the reasons why you prefer that hospital? - Could explain the steps you followed to access the service? - Who directs you to go to this facility for this service? |
| 1. What are the common practices you have before and now; and underlying reasons? | - Was it by sending via telegram, taking the image in person with CD, or going to the referral site and accessing the full service there? - Why do you use this consultation option? Explain the reasons |
| 1. Where is your preference image consultation facility? | - Private imaging clinics, government referral hospitals? - Describe the reasons why you prefer them? |
| 1. How do you evaluate the current medical imaging consultation practice? | - Could you explain it in terms of time and accessibility? - How do you see the affordability of the service in terms of cost? - Are you confortable with the current referral consultation approach? |
| 1. In your view, in the future, what would be the cost effective way to improve the medical imaging service delivery? | - Could explain reasons for your recommendations? - For who you are recommending it |

Thank you for answering all our questions!!!

Perhaps you have an idea that we have overlooked. Is there anything else you'd like to tell me/us about your institutional delivery experience?
