# Supplementary material for: Medical imaging consultation practices and challenges at public hospitals in the Amhara regional state, Northwest Ethiopia: a descriptive phenomenological study
Source: BMC Health Serv Res. 2023 Jul 24;23:787. doi: 10.1186/s12913-023-09652-9 (PMC10367423; doi:10.1186/s12913-023-09652-9)
Supplement: Supplementary file 3 — Additional file 3. Codebook. [file 12913_2023_9652_MOESM3_ESM.docx]

**Title of the manuscript**: Medical Imaging Consultation Practices and Challenges at Public Hospitals in Amhara Regional State, Northwest Ethiopia: A Qualitative Study: Code-book (additional file 3)

| **Code Book** | | | |
| --- | --- | --- | --- |
| **Theme** | **Subthemes** | **Code** | **Code Definition** |
| Medical Imaging service delivery practice |  | Onsite service | - If the radiology image consultation service is provided solely by the onsite physicians /or - When radiologists physical visit primary hospitals to offer radiology service upon invitation |
|  |  | Referral consultation | - It is an image consultation service from a radiologist at a referral hospital - It is the practice where there is an in-person consultation to referral hospitals or private clinics for better management or image interpretation services |
| Image consultation options |  | Softcopy | It is a kind of consulting the digital X-ray image to radiologists via technologies (i.e. via creating telegram channel, coping the X-ray image to secondary storage media or capturing the image by smart phones) |
|  |  | Hardcopy | It is a kind of consulting radiologist via printed image films |
| Advantages and disadvantages of image consultation options |  | Failure of imaging equipment | Dysfunction of imaging equipment |
|  |  | Lack of information completeness | The information submitted is not enough for radiologists to put their final clinical report |
|  |  | Poor image quality | Compromised image quality due to re-capturing via smart phone from computers, poor image handling |
|  |  | Re-imaging | Providing image for the second or third time by disregarding the previous image due to poor quality, scratch, any damage… |
|  |  | Zooming issue | Issues related to unable to zoom in and zoom out as of radiologists desire (lack of viewing the image from different angles) |
|  |  | Susceptible for damage | CDs and film prints are susceptible for easily damage due to poor handling |
|  |  | Demanding physical presence | Image activities such as ultrasound is not suitable for consultation via consulting options |
|  |  | Lack of responsibility | A free service who has no responsible body to respond the service on time |
|  |  | Delayed response time | It is the long response time elapsed to receive their clinical report from radiologists |
|  |  | Knowledge sharing | Enables consulting physicians to share knowledge and experience from radiologists |
| Challenges during radiology service delivery | Organizational factors | Delayed maintenance | Unable to get timely maintenance when the digital equipment encounter a technical problem |
|  |  | Hospital’s regulation | It is the rules and regulations of the hospital how and when to offer radiology service |
|  |  | Frequent power interruption | frequent power on and off |
|  |  | Shortage of imaging equipment | shortage of imaging equipment such as X-Ray, MRI, Ultrasound, CT Scan |
|  |  | Failure of imaging equipment | The dysfunction of any imaging equipment because of natural and manmade faults |
|  |  | Lack of transportation /ambulance | unable to access either public or private transportation services while patients are referred for consultation service |
|  |  | Lack of ICT | Considers the shortage of ICT related infrastructures like internet, local area network,… |
|  |  | Lack of technical skill | Lack of skill either to operate or maintain digital imaging equipment |
|  |  | Shortage of imaging films | It is the shortage/stock out of imaging films |
|  |  | Shortage of infrastructure | Shortage of space for putting imaging equipment |
|  |  | Service inaccessibility | Unable to get the service up on the demand of the community |
|  |  | Capacity building | Unable to access onsite, and off-site short-term and long-term trainings |
|  | Time related factors | Long waiting time/ delayed response | It is the extended time waste to receive radiology service |
|  |  | Long appointment period | It is the high number of patient flow above the hospital standard |
|  |  | High volume of referral overloads | It is the high number of patient flow above the hospital standard |
|  | **Human factors** | Shortage of professionals | Limited number of radiologists and medical-technologists |
|  |  | Shortage of technical experts | Shortage of experts which are capable to maintain imaging equipment |
|  |  | Lack of willingness | Unable to provide technical support, share knowledge, experience to imaging technicians |
|  |  | Lack of awareness | Low level of knowledge about the radiology consultation transportation options |
|  |  | Professional commitment | Commitment of radiologists to give timely image interpretation service up on request |
|  |  | Low patient satisfaction | Dissatisfaction of patients during their stay in the hospital because of the delayed radiology service |
|  |  | Lack of trust | Unable to build trust on image transportation options such as CD |
|  |  | Lack of feedback | Unable to get any feedback be it oral or written from consulted radiologists |
|  |  | Negative perception | It is the perception of patients towards the image transportation options |
|  |  | Image transportation preference | It is the preference of patient/physicians from the image transportation options during their consultation) |
|  |  | Preference of health facility | It is the patients preference based on the hospital regulation to accommodate referral cases, and cost re-imbursement |
|  | **Financial factors** | Shortage of budget | It is the lack of budget to securely provide the radiology service. Or it could be minimum budget allocation |
|  |  | Lack of incentives | It is the shortage of budget to incentivize professionals for the extra workload |
|  |  | Shortage of accommodation cost | (it is a cost related challenge for patients and care givers associated with transportation, bed and food |
|  |  | Shortage of cost for consumables | It is the shortage of budget to buy and avail print films and CDs) |
|  |  | High service cost | It is the cost associated with the imaging service provision) |
| **Challenge overcoming mitigations** |  | Option switching | Switching from one image transportation option to another while encountering shortage/stock out in the market |
|  |  | Referring for better management | Referring pts. looking for better management at the referral health facilities |
|  |  | Awareness creation | Creating awareness for doctors about the consequence of unnecessary image requests |
|  |  | Prioritization | Giving priority for departments based on their patient case level |
|  |  | Service provision by appointment | Entertaining patients based on their date of appointment) |
|  |  | Expert consultation | Conducting consultation seeking for technical, capacity building and financial support from governmental organizations like university teaching hospitals and NGOs |
| Future recommendations for service improvement |  | Creating accessibility | Availing the radiology onsite service as per the demand of the community at their nearby health facility |
|  |  | Technology Assisted | Implementing technology such as teleradiology to improve the radiology service delivery |
|  |  | Flexibility of hospital regulations | Amending the hospital’s rules and regulations to accommodate referral patients |
|  |  | Capacity building | Increase the number of professionals either short-term or long-term capacity building activities |
|  |  | Allocating adequate budget | Securing budget to successfully implement technology such as teleradiology |
|  |  | Incentivizing professionals | Encouraging deserving professionals based on their work overloads |
|  |  | Complying with hospital requirements | Consulting hospitals should adhere the rules and regulations of the referral hospitals |
|  |  | Availing infrastructure | Ensuring ICT infrastructure of the public health facilities with networking and internet; availing imaging equipment such as X-ray, ultrasound, MRI and CT scan to effectively provide radiology service |
|  |  | Unnecessary prescriptions | Avoiding unnecessary image request to save other patients service waiting time |
|  |  | Improving referral system | Creating strong referral system that will consider the demand of the community and number of professionals on the field) |
|  |  | Health insurance | Implementing the health insurance coverage to all parts of the kebeles (the lowest administrative unit) |
