# Supplementary material for: Medical imaging consultation practices and challenges at public hospitals in the Amhara regional state, Northwest Ethiopia: a descriptive phenomenological study
Source: BMC Health Serv Res. 2023 Jul 24;23:787. doi: 10.1186/s12913-023-09652-9 (PMC10367423; doi:10.1186/s12913-023-09652-9)
Supplement: Supplementary file 4 — Additional file 4. COREQ guideline. [file 12913_2023_9652_MOESM4_ESM.docx]

Title of the manuscript: Medical Imaging Consultation Practices and Challenges at Public Hospitals in the Amhara Regional State, Northwest Ethiopia: A Qualitative Study

The consolidated criteria for reporting qualitative research (COREQ) guidelines (Additional file 4)

| **No. Item Guide** | **questions/description** | **Reported on Section** |
| --- | --- | --- |
| **Domain 1: Research team and reflexivity** | | |
| Personal Characteristics | | |
| 1. Interviewer/facilitator | Which author(s) conducted the interview or focus group? | Araya Mesfin  Biniyam Tilahun  Tesfahun Melese  Yonathan Gebreworld  Lemma Derseh  Monika Knudsen  Shegaw Anagaw |
| 2. Credentials | What were the researchers’ credentials? E.g. PhD, MD | AM: MPH, PhD candidate BT: PhD TM: PhD  YG: MD+ LD: PhD  MK: Professor  SA: PhD |
| 3. Occupation | What was their occupation at the time of the study? | AM, BT and TM are researchers in the field of health informatics.  YG is researcher and senior specialist in the field of Radiology  LD is a researcher and biostatistician in the department of Epidemiology and Biostatistics  MK is a senior researcher in mental health digitalization  SA: A researcher in the department of Management Information Systems |
| 4. Gender | Was the researcher male or female? | One of the researchers is female |
| 5. Experience and training | What experience or training did the researchers have? | The researchers have vast experience in qualitative and quantitative research methods. All of them have experience in research and have published their work in international reputable journals |
| Relationship with participants | | |
| 6. Relationship established | Was a relationship established prior to study commencement? | No! The author didn’t have any relationship before study commencement. |
| 7. Participant knowledge of  the interviewer | What did the participants know about the researcher? e.g. personal goals, reasons for doing the research | The consent form and information sheet was presented to participants about the study objectives, and the purpose of the research before the interview started. |
| 8. Interviewer characteristics | What characteristics were reported about the interviewer/facilitator? e.g.  Bias, assumptions, reasons and  interests in the research topic | Reflexivity (Page: 7) |
| Domain 2: study design | | |
| Theoretical framework |  |  |
| 9. Methodological orientation  and Theory | What methodological orientation was stated to underpin the study? e.g. grounded theory, discourse analysis, ethnography, phenomenology, content analysis | Methods- Study design and study period section (Page 4) |
| Participant selection | | |
| 10. Sampling | How were participants selected? e.g. purposive, convenience, consecutive, snowball | Methods: Sample size and sampling techniques (Page 5) |
| 11. Method of approach | How were participants approached? e.g. face-to-face, telephone, mail, email | Methods: Data collection techniques (Page: 6) |
| 12. Sample size | How many participants were in the study? | Methods: Sample size and sampling procedures (Page 5) |
| 13. Non-participation | How many people refused to participate or dropped out? Reasons? | Methods: Data collection tools and procedures (Page: 7) |
| Setting | | |
| 14. Setting of data collection | Where was the data collected? e.g. home, clinic, workplace | Methods: Study setting (Page 5) |
| 15. Presence of nonparticipants | Was anyone else present besides the participants and researchers? | No! Methods: Data collection tools and procedures (Page: 7) |
| 16. Description of sample | What are the important characteristics of the sample? e.g. demographic data, date | Methods: Participant characteristics (Page 6) |
| Data collection | | |
| 17. Interview guide | Were questions, prompts, guides provided by the authors? Was it pilot tested? | Methods: Data collection tools and procedures (Page: 6-7) |
| 18. Repeat interviews | Were repeat inter views carried out? If yes, how many? | No! Methods: Participant selection (Page 6) |
| 19. Audio/visual recording | Did the research use audio or visual recording to collect the data? | Methods: Data collection tools and procedures (Page: 6-7) |
| 20. Field notes | Were field notes made during and/or after the interview or focus group? | Methods: Data collection tools and procedures (Page: 7) |
| 22. Data saturation | Was data saturation discussed? | Methods: Sample size and sampling procedures (Page 5) |
| 23. Transcripts returned | Were transcripts returned to participants for comment and/or correction? | Methods: Data Analysis (Page 8) |
| Domain 3: analysis and findings | | |
| Data analysis |  |  |
| 24. Number of data coders | How many data coders coded the data? | Methods: Data Analysis (Page 8) |
| 25. Description of the coding  tree | Did authors provide a description of the coding tree? | Methods: Theme emergence and development (Page 8) |
| 26. Derivation of themes | Were themes identified in advance or derived from the data? | Methods: Data Analysis (Page 8) |
| 27. Software | What software, if applicable, was used to manage the data? | Methods: Data Analysis (Page 8) |
| 28. Participant checking | Did participants provide feedback on the findings? | Methods: Data analysis (Page: 8) |
| Reporting | | |
| 29. Quotations presented | Were participant quotations presented to illustrate the themes/findings? | Results |
| 30. Data and findings  consistent | Was there consistency between the data presented and the findings? | Discussion |
| 31. Clarity of major themes | Were major themes clearly presented in the findings? | Results |
| 32. Clarity of minor themes | Is there a description of diverse cases or discussion of minor themes? | Results |
